# Short-acting urapidil compared to long-acting phenoxybenzamine in the management of pheochromocytoma

**DOI:** 10.1007/s00423-025-03627-6

**Published:** 2025-02-11

**Authors:** A. Feld, I. Mintziras, S. Wächter, M. Zentgraf, D. K. Bartsch, F. Czubayko, K. Holzer

**Affiliations:** 1 Department of Visceral, Thoracic and Vascular Surgery, Philipps-University Marburg, University Hospital Marburg, Baldingerstrasse, 35043 MarburgMarburg, Germany; 2 Department of Anaesthesiology and Intensive Care Medicine, Philipps-University Marburg, Marburg, Germany; 3 Institute of Pharmacology, Philipps-Universität Marburg, Marburg, Germany

**Keywords:** Urapidil, Phenoxybenzamine, Pheochromocytoma, Surgery, Hemodynamic instability score, Alpha-adrenoceptor blockade

## Abstract

**Purpose:**

In patients with pheochromocytoma current guidelines recommend preoperative alpha-adrenoceptor blockade with selective or nonselective antagonists for at least 7–14 days. To date, no information exists about orally administered urapidil retard, a short-acting selective antagonist.

**Methods:**

The medical records of consecutive patients with pheochromocytoma between 2010 and 2023 were reviewed. Patients received phenoxybenzamine between 2010 and 2017, intravenous urapidil was given between 2017 and 2019. Orally administered urapidil retard has been used from 2019 until present.

**Results:**

Forty-nine patients with pheochromocytomas were included. Twenty-six patients received orally administered long-acting phenoxybenzamine and 23 patients were pretreated with short-acting intravenous (n = 8) or orally administered urapidil (n = 15). Treatment prior to surgery was significantly shorter with intravenously (3 days (IQR, 3–4), *p* = 0.015) or orally administered urapidil (2 days (IQR 2–3), *p* = 0.003) compared to phenoxybenzamine (7 days (IQR, 4–10)). Side effects were more often in the phenoxybenzamine group (17/26 vs 6/23, *p* = 0.02). The modified hemodynamic instability (HI) score was low and there was no significant difference between patients treated with phenoxybenzamine and those treated with intravenous or oral urapidil (29 (IQR 18.5–38); 26 (IQR 18–42); 31 (IQR 15–36) ns). No 30-day postoperative mortality or cardiovascular complications occurred in any of the three groups. The postoperative hospital stay was significantly shorter in the orally administered urapidil group compared to the phenoxybenzamine group (3 days (IQR 3–5)) vs 4 days (IQR 4–5)), *p* = 0.04).

**Conclusion:**

Oral pretreatment with urapidil retard is well tolerated for patients with pheochromocytoma, enabling a safe intra- and postoperative course.

## Introduction

Pheochromocytoma are neuroendocrine tumors arising from chromaffin cells in the adrenal medulla and producing a variety of catecholamines (epinephrine, norepinephrine, and dopamine). Major complaints of patients are sustained or paroxysmal hypertension and arrhythmias. They are at risk of myocardial infarction, cardiomyopathy, and stroke. Surgery and total or subtotal adrenalectomy offer definitive cure for these patients and represent the treatment of choice. Current international guidelines recommend α-adrenergic receptor blocker treatment for 7–14 days [1, 2].

For decades the noncompetitive, long-acting alpha-1 and alpha-2 -adrenoceptor antagonist phenoxybenzamine (Dibenzyran®) was the drug of choice for blockade of pheochromocytoma prior to surgery. Disadvantages of phenoxybenzamine are a substantial incidence of side effects, e.g. nasal congestion, central sedation, syncope, reflex tachycardia, and orthostatic hypotension. The noncompetitive and irreversible action of phenoxybenzamine results in a prolonged duration of action, even in the postoperative setting [3].

The short-acting, competitive and selective alpha-1 adrenoceptor antagonist urapidil (Ebrantil®) has been shown to be as good as phenoxybenzamine. Intravenous urapidil can safely be administered on a normal ward without putting patients at risk [4]. No significant difference was seen in the intraoperative hemodynamic course in 30 patients pretreated with intravenous urapidil or phenoxybenzamine [5]. Already in 2004, Tauzin-Fin et al. published data on 18 patients with pheochromocytoma receiving continuous intravenous infusion of urapidil 10–15 mg/h for 3 days before surgery and until adrenalectomy. It was demonstrated that perioperative intravenous urapidil is a safe and effective alternative during surgical management of pheochromocytoma [6].

Three days of continuous intravenous administration of urapidil prior to surgery is quite uncomfortable for patients. After consultation with our pharmacologist in Marburg we decided to change from intravenous treatment with urapidil to oral medication (urapidil retard, half-life around 4.7 h). The aim of our study of consecutive patients with pheochromocytoma was to demonstrate that urapidil is as safe as phenoxybenzamine prior to surgery and that oral urapidil retard is a good alternative to intravenous urapidil.

## Material and methods

This was a single center study carried out at the Department of Visceral-, Thoracic- and Vascular Surgery of University Hospital Marburg between January 2010 and June 2023. All consecutive patients scheduled for surgery because of pheochromocytoma were retrospectively reviewed. No ethical concerns were raised by the Ethics Committee of University Hospital Marburg (RS 22/42). Inclusion criteria were diagnosis of unilateral or bilateral adrenal mass (CT scan or MRI), elevated free plasma (nor)-metanephrine and/or increased fractionated metanephrines in 24-h urinary excretion. Proven pheochromocytoma in the final histological report was a mandatory requirement for patient enrolment.

Patients received oral phenoxybenzamine between January 2010 and October 2017 (group 1), whereas intravenous urapidil was used between October 2017 and December 2019 (group 2). Orally administered urapidil retard has been used from January 2020 until the present (group 3). Group 1 patients were admitted to hospital on average one week prior to surgery and oral phenoxybenzamine was administered in ascending dosage depending on clinical symptoms. Group 2 patients were admitted for intravenously administered urapidil three days prior to surgery and the infusion was given in ascending dose (day 1: 5 mg/h, day 2: 10 mg/h and day 3: 15 mg/h). If there was no significant drop in blood pressure, intravenous urapidil was continued until adrenalectomy. Orally administered urapidil retard (group 3) was started in-hospital with a standard dose of 120 mg/d po (4 × 30 mg) three days prior to surgery and the dose was increased up to a dose of 260 mg/d the day before surgery. The last urapidil retard tablet (30 mg) was given three hours before surgery.

All data were extracted from the electronic medical records (ORBIS information system). Besides demographic data (age, gender, BMI, ASA classification, germline mutation), tumor diameter, plasma or urine catecholamine level were evaluated. Additionally prescribed antihypertensive drugs were assessed for all patients before admission. On admission, clinical symptoms attributed to pheochromocytomas and side effects of phenoxybenzamine and urapidil were recorded. After admission and during the entire pretreatment period, blood pressure and heart rate were measured three times daily.

An arterial line was placed in all patients directly prior to surgery. Hemodynamic management was performed using a standardized operating procedure. Baseline systolic, highest systolic, and lowest mean arterial pressure, duration of systolic arterial pressure episodes > 160 mm Hg and episodes of mean arterial pressure < 60 mm Hg were assessed. Intraoperatively, the intravenous dose of urapidil or sodium nitroprusside was individually increased, if systolic arterial pressure was exceeded. Following pheochromocytoma resection, hypotension episodes (mean arterial pressure < 60 mmHg) were treated with volume administration and with the vasopressor norepinephrine.

The standard surgical technique was a transperitoneal laparoscopic approach in a lateral position. Open adrenalectomy was performed in a supine position. After surgery, patients were monitored in the recovery room and thereafter on a normal ward or, if necessary, in an intermediate or intensive care unit.

The primary intraoperative outcome of all patients was the modified hemodynamic instability (HI) score. Introduced in 2019 by Buitenwerf E et al., the hemodynamic instability score is a validated score quantifying the degree of hemodynamic instability [7]. The HI score comprises different intraoperative variables, e.g. hemodynamic parameters (systolic blood pressure (SBP), mean arterial pressure (MAP)) as well as therapies (e.g. amount of fluid or vasoactive drugs administered). A higher HI score indicates increasing hemodynamic instability of the patient. Published median values are 44 points in low-risk surgery, and 59 points in high-risk surgery [7]. Buitenwerf et al. modified the HI score when using the score in patients with pheochromocytoma including the dosages of vasodilating drugs and ß-adrenergic receptor blockers [8].

### Statistical analysis

Continuous variables are expressed as medians with interquartile ranges (IQR), categorial variables are presented as proportions. Quantitative variables were compared using the Student´s t-test, or Mann–Whitney U test and qualitative variables using the chi-square test or Fisher´s exact test as appropriate. All reported probabilities values (p-values) are based on the two sided tests, the level of statistical significance was set at p < 0.05. Analyses were performed using SPSS 23.0 (IBM Corp. Released 2015. IBM SPSS Statistics for Windows, Version 23. Armonk, NY: IBM Corp.) and R (version 4.2.1).

## Results

A total of 49 patients (29 female, 20 male) were enrolled in the study. Diagnosis of pheochromocytoma was confirmed by the final pathology result in all patients. Twenty-six patients (15 female, 11 male) were pretreated with orally administered phenoxybenzamine. Twenty-three patients (14 female, 9 male) were pretreated with orally (n = 15) or intravenously (n = 8) administered urapidil.

The mean duration of pretreatment prior to surgery was 7 days (IQR, 4–10) in group 1, 3 days (IQR 3–4) in group 2, and 2 days (IQR, 2–3) in group 3. Patients received a maximum daily dose of 123 ± 63 mg phenoxybenzamine (1.82 ± 1.1 mg/kg bw). Group 2 patients received a maximum daily dose of 3.91 ± 1.06 mg/kg bw of intravenous urapidil. Group 3 patients received a maximum daily dose of 3.72 ± 1.14 mg/kg bw of oral urapidil retard.

The baseline characteristics of all patients are presented in Table 1 and did not significantly differ between the groups.

**Table 1:** Patient characteristics in all groups (median (IQR))

	Group 1 phenoxybenzamine n = 26	Group 2 urapidil (iv) n = 8	Group 3 urapidil retard (po) n = 15	p-value
Male:female ratio	11:15	3:5	6:9	1
Age (years)	54 (47—65)	52 (48—62)	54 (40 – 64)	0.94
BMI (kg/m2)	22.9 (21.3 −25.7)	25.6 (24.5—28.9)	24.5 (20.3 −26.8)	0.20
Symptoms (yes/no)	16/10	5/3	9/6	1
ASA-classification				0.42
I, n	0	0	0	
II, n	10	4	8	
III, n	13	3	7	
IV, n	0	1	0	
Nd,n	3	0	0	
Familial background				0.11
Yes, n	7	1	4	
No, n	8	1	8	
Not assessed n	11	6	3	
Tumor diameter (cm)	4.1 (3—5.8)	4.8 (2.3—5.6)	4.1 (3—5.8)	0.71
Plasma-free normetanephrine (pg/ml)*1	nd	1655 (1331 −2719)	406 (1331 −2719)	0.44
Plasma-free metanephrine (pg/ml)*2	nd	640 (363 −1170)	114 (72–245)	0.73
Normetanephrine 24 h urine (nmol/d) *3	5831 (2005 −11227)	7636 (5527- 28545)	6895 (4061—7687)	0.38
Metanephrine 24 h urine (nmol/d) *4	3140 (1414 −7230)	10189 (7948 −23681)	2373 (483—25652)	0.17
Surgery				0.47
Laparoscopic, n (%)	21	8	15	
Open n (%)	02	0	0	
Conversion	03	0	0	
Operative time (min)	97 (80—170)	148(118—164)	93 (87—118)	0.054
Anesthesia (min)	213 (185—282)	260 (230—270)	210 (192.5—235)	0.14

ASA-classification = American Society of Anesthesiologists classification, nd = not done

Normal range*1) < 65 pg/ml, *2 < 196 pg/ml, *3 < 2600 nmol/d, * 4 < 1510 nmol/d

**Fig. 1:**
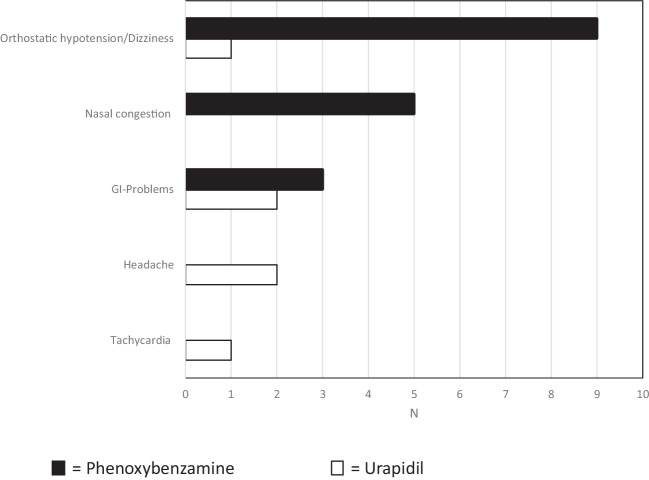
Side effects of patients pretreated with phenoxybenzamine or urapidil (iv and po)

Side effects were described more often in patients with phenoxybenzamine pretreatment (17/26). Besides nasal congestion (n = 5) and gastrointestinal problems (n = 3), dizziness and orthostatic hypotension were described in nine patients. Patients pretreated with urapidil had significantly fewer side effects (6/23) compared to phenoxybenzamine. Apart from headache (n = 2) and gastrointestinal problems (n = 2), dizziness (n = 1) and tachycardia (n = 1) were observed (*p* = 0.02) (Fig. 1).

In the phenoxybenzamine group, laparoscopic pheochromocytoma surgery was performed in 21/26 patients and open adrenalectomy in two patients. Conversion to an open procedure was necessary in three patients. In both urapidil groups, all patients (23/23) were operated laparoscopically. There was no need for blood transfusions in any of the three groups. Intraoperatively, the modified total HI score did not differ between the groups 1–3 (group 1: 29 (IQR 18.5–38) group 2: 26 (IQR 18–42), group 3: 31 (IQR 15–36); ns) (Table 2).

**Table 2:** Modified hemodynamic instability score

	Group 1 phenoxybenzamine n = 26	Group 2 urapidil (iv) n = 8	Group 3 urapidil (po) n = 15	p-value
Maximum SBP (mmHg)	150 (140 – 170)	165 (153—173)	180 (155—208)	0.009
Time SBP > 160 mmHg(%)	0.00 (0.00—3.3)	2.33 (0.00—7.35)	9.09 (0.00—12.5)	ns
Minimum MAP (mmHg)	60 (57—66)	60 (58—64)	60 (57—67)	ns
Time MAP < 60 mmHg(%)	0.00 (0.00—2.76)	0.00 (0.00—0.00)	0.00 (0.00—2.25)	ns
Maximum HR (bpm)	95 (90—114)	105 (97—113)	98 (90—108)	ns
Time HR > 100 (%)	0.00 (0.00—8.6)	1.92 (0.00—7.9)	0.00 (0.00—6.1)	ns
Minimum HR (bpm)	58 (50—64)	55 (51—59)	60 (58—68)	ns
Time HR < 50 bpm (%)	0.00 (0.00—0.00)	0.00 (0.00—0.00)	0.00 (0.00—0.00)	ns
Volume therapy (ml/kg/h)	9 (6.5—11.4)	6.6 (6.3 – 10.1)	9.1 (7.1- 12.3)	ns
Norepinephrine (µg/kg/h)	1.9 (0.2 −4)	2.2 (0.9 −8.6)	1.6 (0.9—2.7)	ns
Phenylephrine (µg/kg/h)	not given	not given	not given	
Vasopressors (other)	7	0	1	
Vasodilators (other)	5	3	7	
Magnesium sulphate (g/kg/h)	not given	not given	not given	
Phentolamine	not given	not given	not given 0	
Esmolol (mg/kg/h)	0.00 (0.00—0.00)	0.00 (0.00—0.00)	0.0038 (0.0038—0.0038)	
ß-adrenergic receptor blocker	5	1	3	
**HI score (total)**	**29 (18.5—38)**	**26 (18—42)**	**31 (15—36)**	**ns**

Data presented as median (Interquartile range)

Abbreviations: HI-score, hemodynamic instability score,: SBP, systolic blood pressure, MAP, mean arterial pressure, HR, heart rate

Vasopressor: (other): Akrinor ®

Vasodilator (other): sodium nitroprusside, clonidine

Maximum intraoperative systolic pressure was higher in group 3 compared to groups 1. (group 1: 150 mmHg (IQR 140–170) group 3: 180 mmHg (IQR 155–208), *p* = 0.009). There was no significant difference in intraoperative volume administration between any of the groups (group 1: 9 ml/kg/h (IQR 6.5–11.4), group 2: 6.6 ml/kg/h (IQR 6.3–10.1), group 3: 9.1 ml/kg/h (IQR 7.1–12.3), ns)). The need for intraoperative vasopressor (norepinephrine) was without significant difference (Table 2).

In the early postoperative course, there were no significant differences between the groups with respect to the occurrence of postoperative hypotension defined as a mean arterial pressure < 60 mmHg or the use of infusions or vasopressor (norepinephrine). Two out of 26 patients in group 1 (7.7%) and two out of eight patients in group 2 (25%) needed norepinephrine postoperatively. In group 3 there was no need for postoperative norepinephrine.

No 30-day postoperative mortality or major cardiovascular complications occurred in any of the groups.

Postoperative hospital stay in the groups 1 and 3 was also significantly different (group 1: 4 days (IQR 4–5) vs. 3 days (IQR 3–5), *p* = 0.04).

## Discussion

Although never tested in a randomized controlled trial (RCT), patient pretreatment with an alpha receptor blocker is considered mandatory according to international guidelines for pheochromocytomas [1, 2]. Preoperative treatment should be initiated as early as 7–14 days before surgery. For a long time, the noncompetitive, long-acting alpha-1 and alpha-2 adrenoceptor antagonist phenoxybenzamine, with a pharmacological half-life of 24 h, was the drug of choice [3]. The well-known disadvantages of phenoxybenzamine are long acting because of irreversibility and a high incidence of reflex tachycardia, central sedation, and orthostatic hypotension [9].

Urapidil is a competitive α1 blocker with a much shorter half-life. The preoperative use of intravenous urapidil was first described in 1996. In a study of seven consecutive patients undergoing pheochromocytoma resection, Steib et al. used intravenous urapidil [10]. In 2004, Tauzin-Fin et al. evaluated 18 patients with pheochromocytoma receiving intravenous infusion of urapidil 10–15 mg/h 3 days before surgery and until adrenalectomy. The authors concluded that perioperative blocking using intravenous urapidil is safe and an effective alternative during surgical management of pheochromocytoma [6]. In 2013, Habbe et al. published data on 30 patients pretreated with intravenous urapidil (n = 11) or phenoxybenzamine (n = 19). No significant difference was seen in the intraoperative hemodynamic course [5]. In 2020, Tauzin-Fin et al. published data on 75 patients with pheochromocytomas pretreated for three days with continuous intravenous infusion of urapidil and with stepwise increase to the maximum tolerated dose [11].

Three days of continuous intravenous urapidil prior to surgery is not comfortable and sometimes even stressful for patients. Starting In 2020, after consultation with our pharmacologist in Marburg, we decided to change from intravenous urapidil treatment to oral medication (urapidil retard) prior to pheochromocytoma surgery. In our study of consecutive patients with pheochromocytoma we demonstrated that continuous intravenous urapidil is safe. In addition, our data reveals for the first time that orally administered urapidil retard is safe too and is a good alternative for pretreatment of patients with pheochromocytomas. Furthermore, pretreatment with intravenous or oral urapidil is much shorter and had significantly fewer side effects compared to phenoxybenzamine. During surgery no significant difference was seen in the hemodynamic instability score following intravenous or oral urapidil pretreatment compared to phenoxybenzamine. No 30-day postoperative mortality or major cardiovascular complications occurred in any of the groups. Postoperative monitoring in the intensive care unit was more common in patients pretreated with phenoxybenzamine compared to both urapidil groups. The postoperative hospital stay was significantly shorter in the orally administered urapidil group compared to the phenoxybenzamine group.

To date, no consensus has been reached on the optimal preoperative regimen for pheochromocytomas. In the United Kingdom, preoperative alpha blockers were reported to be routinely used with a preference for phenoxybenzamine (72%) [12]. In the United States, phenoxybenzamine is no longer the standard agent used for alpha blockade before adrenalectomy for pheochromocytoma. The proportion of patients blocked with phenoxybenzamine decreased from 71.0% in 2008 to 21.2% in 2019. Selective alpha blockers or calcium channel blockers are increasingly used [13].

Only one multicenter randomized controlled trial has been published so far on the pretreatment of patients with pheochromocytoma. Buitenwerf et al. demonstrated recently that the use of phenoxybenzamine compared to the selective alpha-1 adrenergic receptor blocker doxazosin prior to surgery had no significant impact on blood pressure outside the target range (*p* = 0.75) [14].

A recent meta-analysis showed that there was no significant difference between selective or nonselective alpha blockade in pheochromocytoma patients regarding intraoperative systolic blood pressure (SBP) > 160 mm Hg (relative risk (RR) 0.95 [95% CI 0.57, 1.56] *P* = 0·83) and intraoperative vasopressor requirement (RR 1.10 [95% CI 0.96, 1.26] *P* = 0·16). The authors stated that on using phenoxybenzamine instead of selective alpha blocker in patients with pheochromocytoma, no clinical benefit could be ascertained with regard to morbidity and mortality [15].

In recent years the question was raised whether perioperative α-receptor blockade was still necessary for the pretreatment for all patients with pheochromocytoma [16]. For instance, in patients with asymptomatic normotensive pheochromocytoma or small hereditary pheochromocytoma with lower secretion of catecholamines.

Two meta-analyses have been carried out regarding the question preoperative α-blockade versus no blockade before adrenalectomy for patients with pheochhromocytoma [17, 18].

For an experienced team of surgeons and anesthesiologists it is possible to manage unblocked patients with pheochromocytoma safely during anesthesia and surgery. But there is little need to take unnecessary risks, especially in symptomatic patients, when effective preoperative blockade can be done so easily and with such short duration as with oral urapidil retard.

The present study has certain limitations and differences must be interpreted carefully. The single center retrospective study of patients with pheochromocytoma over a long period has a small sample size and consecutive data include a clear bias.

## Conclusion

For the first time we were able to demonstrate that the use of short-acting oral urapidil retard in patients with pheochromocytomas prior to surgery is comfortable and enables a safe intra- and postoperative course. The hemodynamic instability score was low and did not differ significantly when compared to patients pretreated with intravenous urapidil or phenoxybenzamine.

## Data Availability

The data that support the findings of this study are not openly available due to reasons of sensitivity and are available from the corresponding author upon reasonable request.
